# Massachusetts

**Published:** 1897-02

**Authors:** 


*Massachusetts.*—Governor Wolcott, in his annual message to the Legislature, treats of the control and eradication of bovine tuberculosis, refers to the better understanding of the subject from a scientific standpoint, and lays particular stress on the danger when the udder is diseased. Sterilization of all milk is suggested. He refers to those who consider the danger slight when the disease is only in the incipient stage. At present the tuberculin-test is restricted to animals brought in from outside the Commonwealth and at quarantine stations, and may be used after consent in writing obtained of owners of animals within the State and upon animals condemned as tuberculous after physical examination. These restrictions expire by limitation June 1, 1897. He thinks the increased knowledge on the subject and the demands of milk- and meat-consumers will largely contribute to a solution of the problem. The tuberculin-test has his hearty indorsement. He offers two suggestions for consideration: First, Is it right or wise that, as now, the State should pay full value for animals that have reached the most advanced stage of general tuberculosis and udder tuberculosis? Such animals should be brought out by means of a thorough periodic inspection, and slaughtered as being not only worthless but a source of danger to the rest of the herd as well as to the community. At present the owner has no motive to check the disease in its earlier stages. If compensation were graded accerding to the condition of the animal as revealed by the autopsy, the owner would have a direct interest in purging his herd of infected animals before they become worthless. Secondly, he thinks "the commissioners should in any event have sufficient means at their disposal to enable them to test with tuberculin all cattle the owners of which request such inspection."

